# Prevalence of cognitive frailty in older adults with stroke in China: a systematic review and meta-analysis

**DOI:** 10.3389/fneur.2026.1742673

**Published:** 2026-07-08

**Authors:** Yang Li, Zhiyuan Zhang, Yonghua Cai, Yutong Cui, Xuewei Zhan, Xiaoxing Lai, Xiaopeng Huo, Donglei Shi

**Affiliations:** 1 Department of Neurology, Peking Union Medical College Hospital, Chinese Academy of Medical Sciences & Peking Union Medical College, Beijing, China; 2 Department of Nursing, Peking Union Medical College Hospital, Chinese Academy of Medical Sciences & Peking Union Medical College, Beijing, China; 3 Department of International Medical Services, Peking Union Medical College Hospital, Chinese Academy of Medical Sciences & Peking Union Medical College, Beijing, China

**Keywords:** cognitive frailty, meta-analysis, older adults, prevalence, stroke

## Abstract

**Objective:**

Stroke is a leading cause of disability and mortality in older adults. Post-stroke cognitive frailty (CF) increases the risk of adverse outcomes. However, evidence regarding the prevalence of CF remains limited and inconsistent. This study aims to systematically evaluate the prevalence of CF among older adults with stroke in China and identifies factors associated with variation in prevalence, thereby informing future research directions and potential clinical considerations.

**Methods:**

We searched PubMed, Embase, Web of Science, Cochrane Library, China Biomedical Literature Database (CBM), China National Knowledge Infrastructure (CNKI), VIP Database, and Wanfang Database for relevant studies reporting the prevalence of CF in Chinese older adults with stroke. The search covered the period from each database’s inception to November 2024. Eligible studies were assessed for quality, and data were extracted. All analyses were performed using Stata 18.0 software.

**Results:**

Four Chinese studies involving 1,337 participants were included. Meta-analysis revealed a pooled prevalence of CF among older adults with stroke of 33% (95% CI: 28–39%). Subgroup analysis indicated a significantly higher prevalence of CF in stroke patients aged ≥80 years compared to those aged 60–79 years (*p* < 0.05). Furthermore, the prevalence was significantly higher among those who consumed alcohol than among non-consumers (*p* < 0.05). Notably, subgroup estimates for patients with malnutrition and depression were markedly high, at 47% (95% CI: 34–60%) and 62% (95% CI: 53–71%), respectively.

**Conclusion:**

Available evidence suggests that the prevalence of CF among older adults with stroke in China appears to be substantial, with significant variations associated with alcohol consumption and advanced age. The particularly high prevalence estimates in patients with malnutrition or depression warrant increased clinical attention and further investigation into their relationship with post-stroke CF. Caution is warranted given the limited evidence base (*n* = 4), high heterogeneity, and exclusively hospital-based Chinese setting; larger studies are needed to confirm these observations. Early identification and evidence-based interventions targeting CF in this population may be crucial to mitigating adverse health outcomes pending further validation.

**Systematic review registration:**

This systematic review was prospectively registered with PROSPERO (registration number: CRD42025643159).

## 1 Introduction

China is experiencing rapid population aging, with over 300 million people aged 60 years and above, which is projected to reach about one-third of the total population by 2050 (1). Concurrently, the prevalence and incidence of stroke increase with age, making older adults the most affected demographic (2). Stroke survivors often suffer from complications like neurovascular damage, dysphagia, speech impairment, and hemiplegia. These sequelae can lead to malnutrition, reduced physical activity, and declines in both physical and cognitive function (3, 4).

Cognitive frailty (CF) is a clinical syndrome defined by the co-occurrence of physical frailty and mild cognitive impairment, excluding Alzheimer’s disease and other dementias (5). As an emerging post-stroke complication, CF is linked to an increased risk of adverse health outcomes, including disability, dementia, and mortality, warranting attention from clinicians and researchers (6, 7).

Existing literature, both domestic and international, has predominantly examined cognitive impairment and physical frailty in older adults with stroke as separate entities. Research dedicated specifically to CF remains scarce. This limited focus may underestimate the complex interplay between cognitive decline and frailty, as well as their combined effect on the rehabilitation trajectory of older adults recovering from stroke. Furthermore, inconsistencies in assessment tools, diagnostic criteria, and study populations have led to considerable variability in reported rates of CF within this group.

Therefore, this study aims to systematically evaluate the prevalence of CF among older adults with stroke, thereby offering an evidence base to guide the early identification and prevention of CF in this population.

## 2 Methods

### 2.1 Literature inclusion and exclusion criteria

*Inclusion criteria*: (1) Participants met the diagnostic criteria for stroke as defined by the 2018 Chinese Guidelines for the Diagnosis and Treatment of Acute Ischemic Stroke (8) and were aged ≥60 years, or where data for the ≥60-year subgroup could be separately extracted. (2) Studies that explicitly defined CF and specified its diagnostic criteria (5, 9). (3) Studies were conducted in mainland China. (4) Study designs were cross-sectional or cohort. (5) Publications were in either Chinese or English.

*Exclusion criteria*: (1) Studies containing erroneous, questionable, or incomplete data that precluded analysis. (2) Studies for which the full text was unavailable or that were duplicate publications. (3) Studies with a sample size of less than 50 participants. (4) Review articles, editorials, or other non-original research publications.

### 2.2 Literature sources and search strategy

This systematic review and meta-analysis was prospectively registered with PROSPERO (registration number: CRD42025643159).

A comprehensive literature search was performed across the following databases: PubMed, Embase, Web of Science, Cochrane Library, CBM, CNKI, VIP Database, and Wanfang Database. The aim was to identify studies reporting the prevalence of CF in older adults with stroke. The search covered the period from database inception to November 2024. Two experienced reviewers independently designed and executed the search strategy to ensure high sensitivity. The search strategy combined controlled vocabulary (e.g., MeSH terms in PubMed, Emtree in Embase) with free-text keywords related to three core concepts: (1) older adults with stroke, (2) cognitive frailty, and (3) prevalence or epidemiology. Boolean operators (AND, OR, NOT) were used to logically combine these concepts and their synonyms. The specific search syntax was meticulously adapted to conform to the rules and functionalities of each individual database. To supplement the electronic search and capture any potentially eligible studies that may have been missed, we also manually screened the reference lists of all included studies and relevant review articles. The complete, database-specific search strategies are provided in Supplementary file 1.

### 2.3 Literature screening and data extraction

A systematic search of multiple Chinese and English databases was performed in accordance with the predefined strategy to identify relevant cross-sectional and cohort studies. Retrieved records were imported into NoteExpress software for management and deduplication. Following training in systematic review methodology, two researchers independently screened the titles and abstracts against the inclusion and exclusion criteria to identify potentially eligible studies. The full texts of these candidate articles were then obtained and assessed in a second screening phase to exclude those that did not meet the criteria.

Data extraction was conducted independently by the same two researchers using standardized form, employing a double-blind approach to minimize bias. Extracted information included the first author’s name, country, publication year, study design, study population, assessment tools used, and the reported prevalence of CF among older adults with stroke. All extracted data were cross-verified to ensure accuracy and consistency. Any discrepancies identified during this process were resolved through discussion or, if necessary, by consultation with a third reviewer to ensure objectivity and reliability.

### 2.4 Quality assessment of the literature

The methodological quality of the included cross-sectional studies was assessed using the quality evaluation checklist recommended by the Agency for Healthcare Research and Quality (AHRQ). This checklist comprises 11 items, each of which can be assigned 1 point, for a maximum possible score of 11. Based on established cut-offs, studies were classified as low quality (0–3), medium quality (4–7 points), or high quality (8–11 points) (10). For cohort studies, the risk of bias was evaluated using the Newcastle-Ottawa Scale (NOS). This scale consists of three domains encompassing a total of eight items. Each item meeting the evaluation criteria is awarded 1 or 2 points, resulting in a maximum achievable score of 9. Studies were subsequently rated as low quality (0–3 points), medium quality, (4–6 points), or high quality (7–9 points) (11).

### 2.5 Statistical analysis

All meta-analyses were performed using Stata 18.0 software. Statistical heterogeneity among the included studies was evaluated using the Cochran’s *Q* test and quantified by the *I*^2^ statistic. An *I*^2^ value of less than 50% was considered indicative of moderate heterogeneity, while a value of 50% or greater denoted high heterogeneity. A fixed-effects model was applied when no significant statistical heterogeneity was observed; otherwise, a random-effects model was used. Although the four included studies used variable combinations of validated frailty and cognitive assessment instruments (Table 1), pooling of effect estimates was deemed methodologically justified. All studies operationalized CF per the International Academy on Nutrition and Aging (IANA)/International Association of Gerontology and Geriatrics (IAGG) operational consensus (5, 9), defined as co-occurring physical frailty and mild cognitive impairment with explicit exclusion of dementia. Each study employed widely validated instruments to capture these two core domains, and a random-effects model was used to account for between-study variability in assessment tools and diagnostic thresholds. Publication bias was assessed using Begg’s test. However, it is noteworthy that this test has low statistical power when the number of included studies is less than five. Therefore, a funnel plot was also generated to allow for visual inspection. For all analyses, a two-sided *p*-value of less than 0.05 was considered statistically significant.

**Table 1:** Basic characteristics of the four included studies.

Author	Region	Source	Study type	CF assessment tools	CF diagnostic criteria	Total sample/CF cases	AHRQ score	NOS score
Pan et al. (14)	Nanjing, Jiangsu, China	Hospital	Cross-sectional study	FRAIL CDR	① FRAIL:≥1 point; ② CDR = 0.5 point; ③ No clinical dementia diagnosis.	305/94	9	
Lin et al. (13)	Hangzhou, Zhejiang, China	Hospital	Cross-sectional study	FRAIL MMSE	① FRAIL: ≥3 points; ② MMSE: Illiterate ≤17 points, Primary ≤20 points, Secondary and above ≤24 points; ③ Excluded clinical dementia diagnosis.	236/83	8	
Hu et al. (12)	Nanjing, Jiangsu, China	Hospital	Cross-sectional study	Edmonton CDR MMSE	① Edmonton: >6 points; ② CDR = 0.5 point; ③ 24 points ≤ MMSE < 27 points.	96/42	8	
Zhao et al. (15)	Tangshan, Hebei, China	Hospital	Cohort study	FFP MMSE	① FFP: ≥3 points; ② MMSE: Illiterate ≤17 points, primary ≤20 points, secondary and above ≤24 points; ③ Excluded clinical dementia diagnosis.	700/271		9

FRAIL, the fatigue, resistance, ambulation, illness, and loss of weight scale; Edmonton, cognitive ability, basic control status, independence, social support holding, drug use, nutrition, mood, control, functional performance scales; MMSE, mini-mental state examination; FFP, fried frailty phenotype; CDR, clinical dementia rating.

## 3 Results

### 3.1 Literature screening process and results

**Figure 1:**
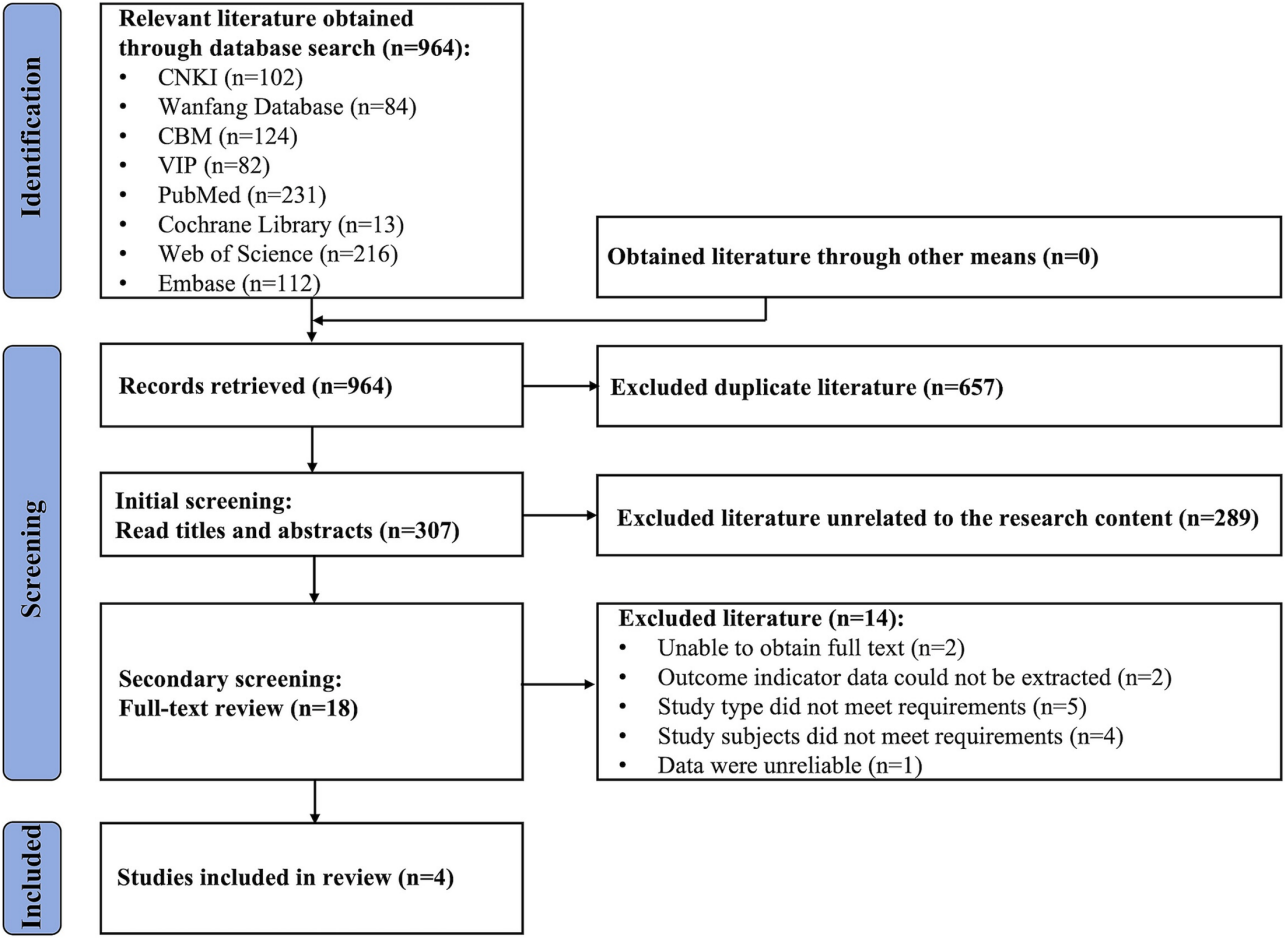
PRISMA-based literature screening flowchart.

The initial database searches yielded a total of 964 records. After a systematic screening process, four studies were ultimately included (12–15). The final set consisted of three cross-sectional studies (12–14) and one cohort study (15). A flowchart detailing the screening process is provided in Figure 1.

### 3.2 Basic characteristics and quality assessment of included studies

The four included studies involved a total of 1,337 older adults with stroke, of whom 490 were diagnosed with CF. All participants were recruited from hospital settings across multiple regions in China, specifically Nanjing (Jiangsu Province), Hangzhou (Zhejiang Province), and Tangshan (Hebei Province). Publication years for the included studies ranged from 2020 to 2024. The basic characteristics of these studies are summarized in Table 1. Regarding methodological quality, all three cross-sectional studies were rated as high quality (12–14), as detailed in Table 2. The single cohort study was also classified as high quality (15). The complete results of the quality assessments are presented in Tables 2 and 3.

**Table 2:** Quality assessment results for the three cross-sectional studies.

Author	①	②	③	④	⑤	⑥	⑦	⑧	⑨	⑩	⑪	Total score (points)	Quality rating
Pan et al. (14)	Y	Y	Y	Y	Y	Y	Y	Y	N	Y	N	9	High
Lin et al. (13)	Y	Y	Y	Y	Y	Y	N	Y	N	Y	N	8	High
Hu et al. (12)	Y	Y	Y	Y	Y	Y	N	Y	N	Y	N	8	High

Y, yes; N, no/unclear. Evaluation items: ① Whether the source of information was clearly defined (e.g., survey, literature review)? ② Were inclusion/exclusion criteria for exposure and non-exposure groups clearly defined or referenced? ③ Was the time frame for identifying patients provided? ④ If not population-based, were the study subjects consecutively included? ⑤ Were assessors blinded to other objective indicators when evaluating subjective measures? ⑥ Were any quality assurance methods described (e.g., repeated measures of key outcomes)? ⑦ Was any explanation provided for excluding certain patients? ⑧ Were methods for assessing and controlling confounding factors described? ⑨ Were missing data explained? ⑩ Were response rates and completeness of data collection summarized? ⑪ If follow-up was involved, was the percentage of incomplete follow-up data or results specified?

**Table 3:** Quality assessment results for the cohort study.

Author	①	②	③	④	⑤	⑥	⑦	⑧	Total score (points)	Quality rating
Zhao et al. (15)	1	1	1	1	2	1	1	1	9	High

The NOS scale uses a semi-quantitative star system, where most items score a maximum of 1 star, except for “comparability between groups,” which can score up to 2 stars. A total score of 9 stars indicates high-quality research. Evaluation items: ① Representativeness of the exposed cohort; ② Selection of the non-exposed cohort; ③ Ascertainment of exposure; ④ Demonstration that the outcome of interest was not present at the start of the study; ⑤ Comparability of cohorts based on design or analysis; ⑥ Assessment of outcome; ⑦ Adequacy of follow-up duration; ⑧ Completeness of follow-up for cohorts.

### 3.3 Meta-analysis of the overall prevalence of CF in older adults with stroke

**Figure 2:**
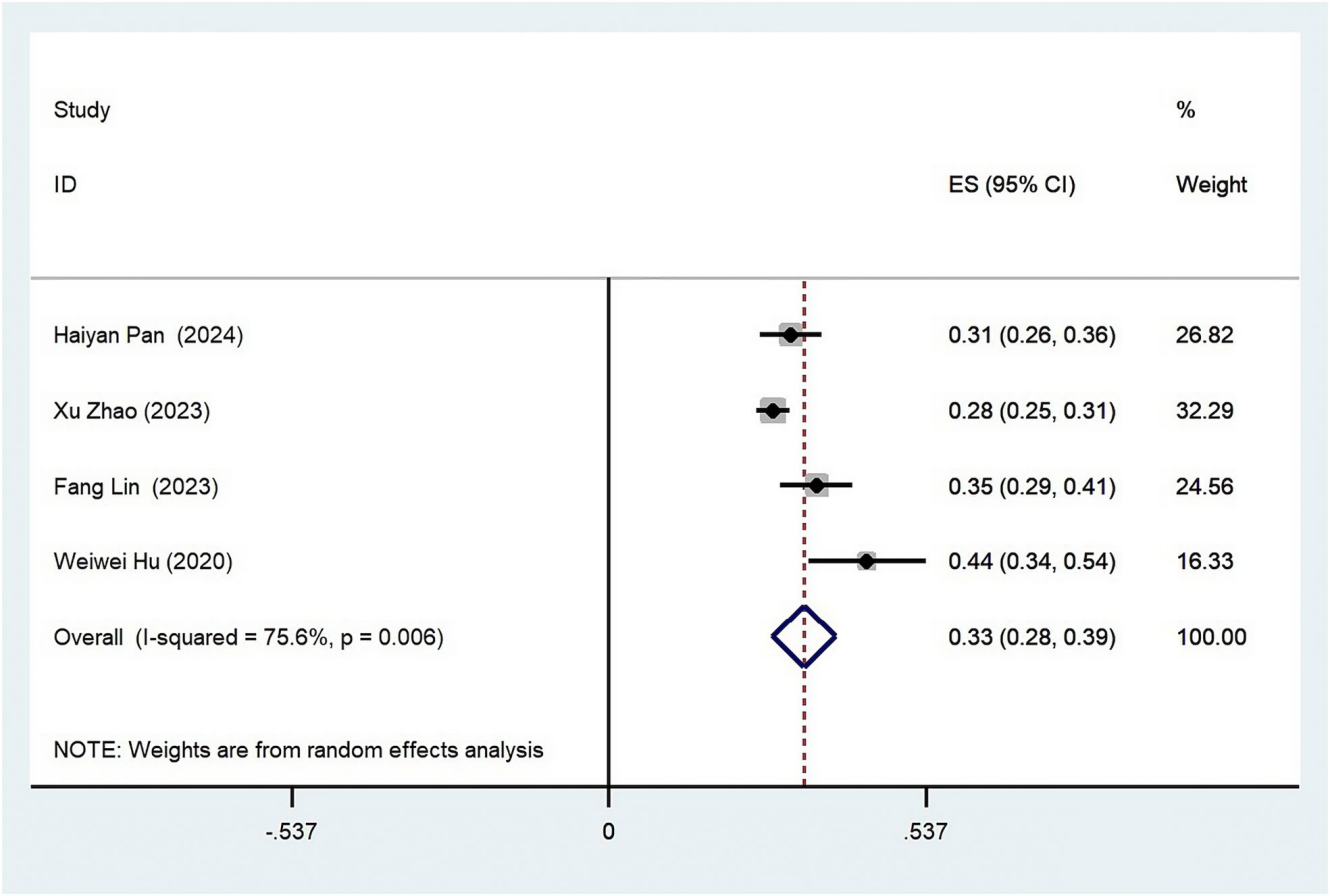
Forest plot of the meta-analysis on the overall prevalence of CF in older adults with stroke.

The prevalence of CF among older adults with stroke reported in the included studies ranged from 27.90 to 43.75%. Heterogeneity testing yielded an *I*^2^ value of 75.6% (*p* < 0.01), indicating significant heterogeneity. Consequently, a random-effects model was applied for the meta-analysis. The combination of high statistical heterogeneity and a small number of included studies (*n* = 4) warrants cautious interpretation of the pooled estimate. The pooled overall prevalence of CF in this population in China was 33% (95% CI: 28–39%), as shown in Figure 2.

### 3.4 Subgroup analysis of CF prevalence in older adults with stroke

**Figure 3:**
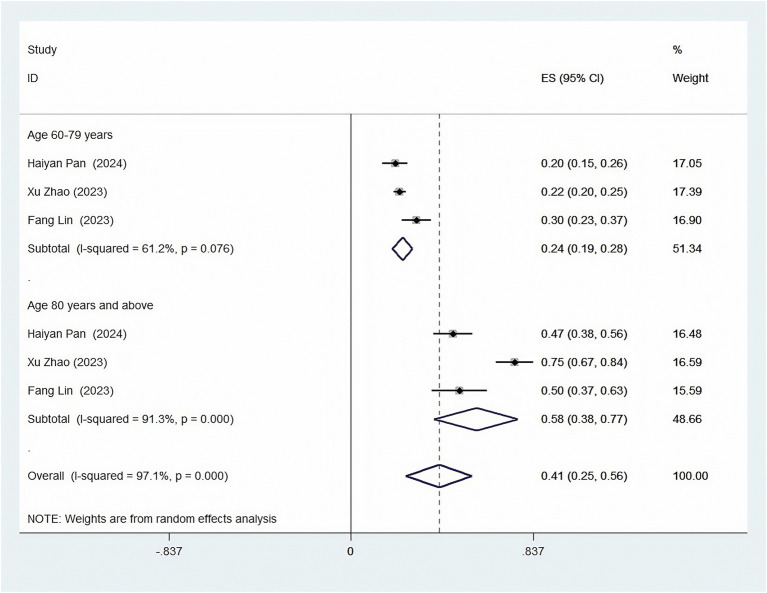
Forest plot of CF prevalence by age in older adults with stroke.

**Figure 4:**
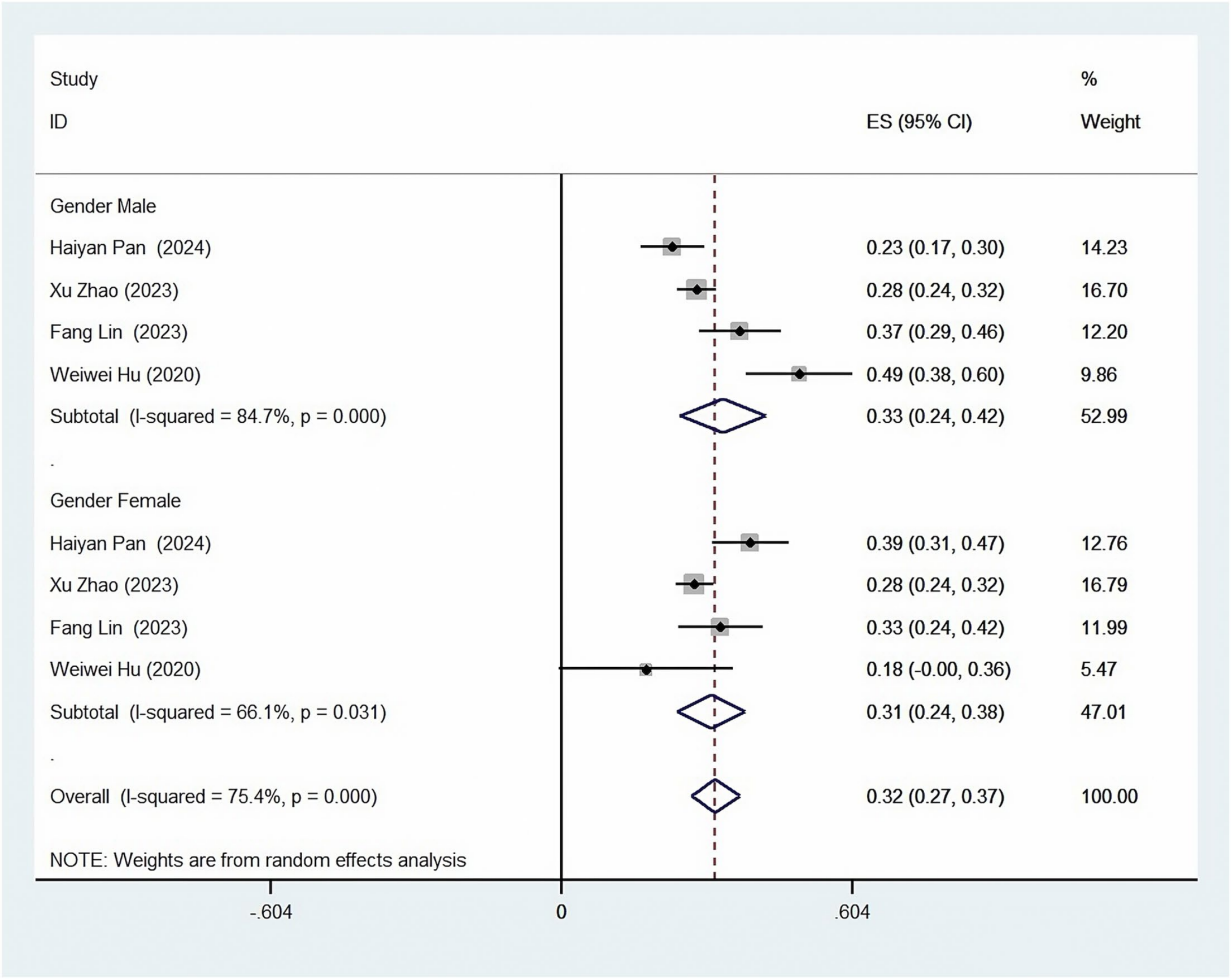
Forest plot of CF prevalence by gender in older adults with stroke.

**Figure 5:**
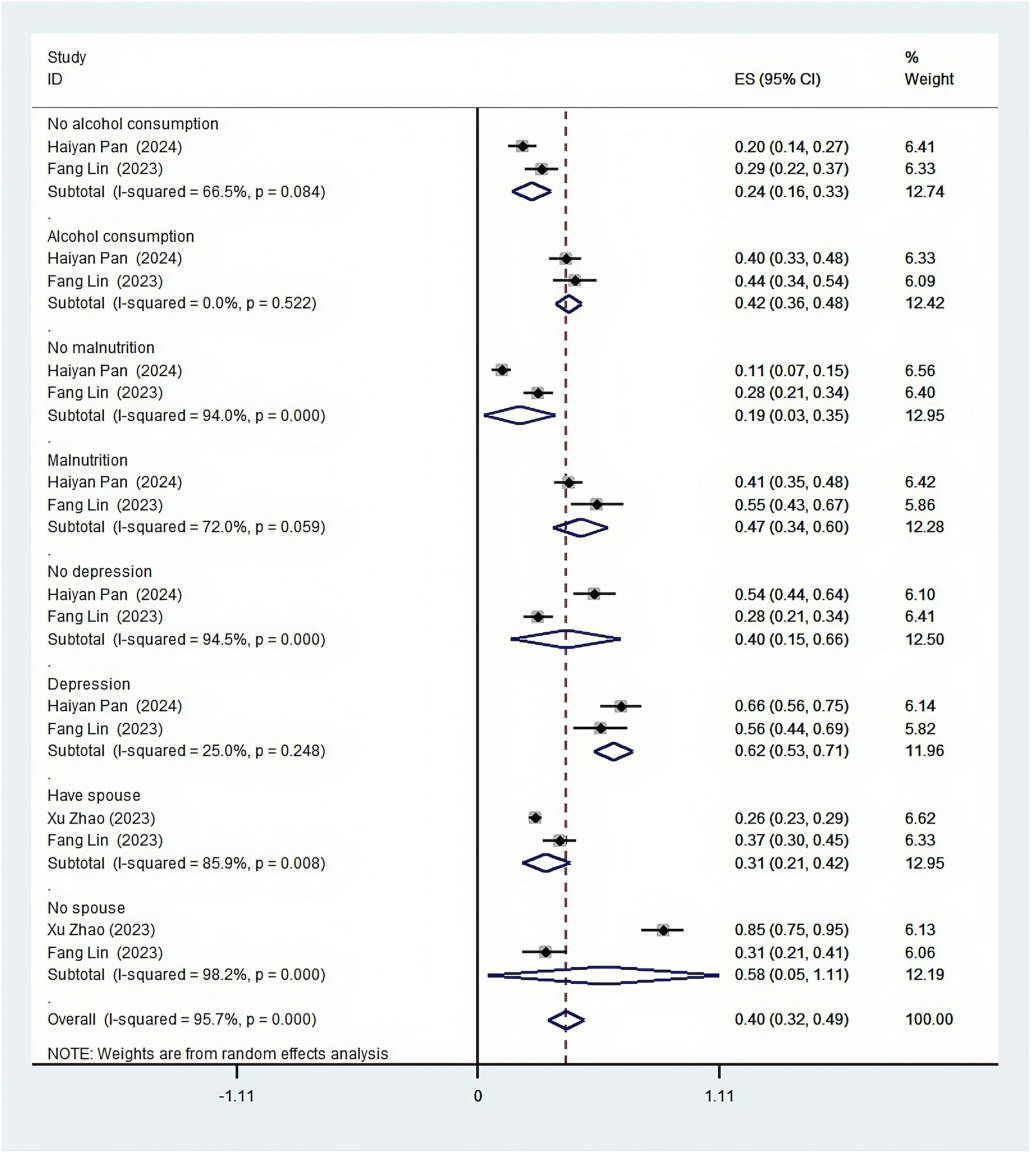
Forest plot of CF prevalence by different risk factors in older adults with stroke.

Subgroup analyses were performed based on age (Figure 3), gender (Figure 4), and other factors, including alcohol consumption, malnutrition, depression, and marital status (Figure 5):

#### 3.4.1 Age

- The prevalence of CF among stroke patients aged 60–79 years was 24% (95% CI: 19–28%).
- The prevalence among those aged 80 years or older was 58% (95% CI: 38–77%). This difference was statistically significant (*p* < 0.05).

#### 3.4.2 Gender

- The prevalence was 33% (95% CI: 24–42%) in male patients.
- The prevalence was 31% (95% CI: 24–38%) in female patients.

#### 3.4.3 Alcohol consumption

- Among non-drinkers, the prevalence was 24% (95% CI: 16–33%).
- Among those who consumed alcohol, the prevalence was 42% (95% CI: 36–48%). This difference was statistically significant (p < 0.05).

#### 3.4.4 Malnutrition

- In patients without malnutrition, the prevalence was 19% (95% CI: 3–35%).
- In patients with malnutrition, the prevalence was 47% (95% CI: 34–60%).

#### 3.4.5 Depression

- In patients without depression, the prevalence was 40% (95% CI: 15–66%).
- In patients with depression, the prevalence was 62% (95% CI: 53–71%).

#### 3.4.6 Marital status

- Among patients who had a spouse, the prevalence was 31% (95% CI: 21–42%).
- Among patients without a spouse, the prevalence was 58% (95% CI: 5–100%).

### 3.5 Publication bias analysis

**Figure 6:**
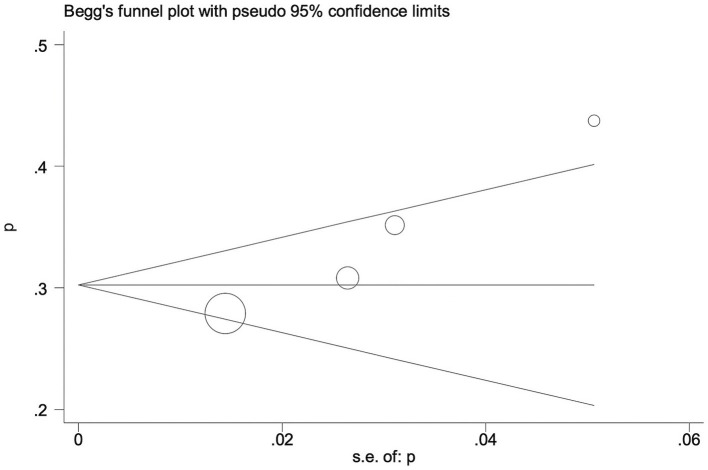
Begg’s funnel plot for bias analysis.

**Figure 7:**
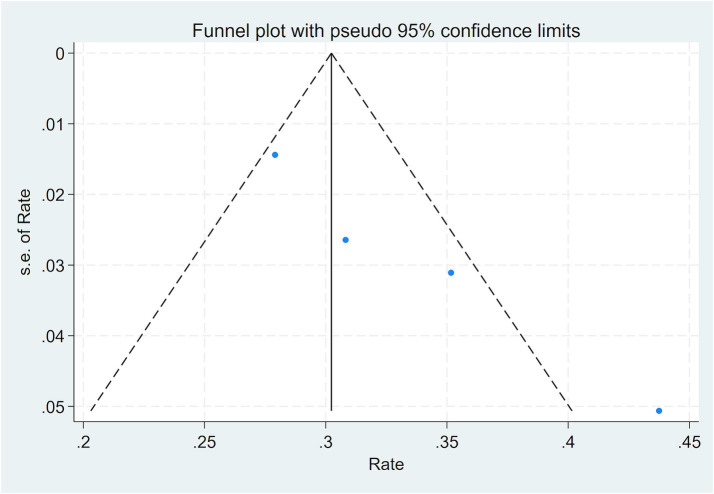
Funnel plot for overall prevalence of CF.

**Figure 8:**
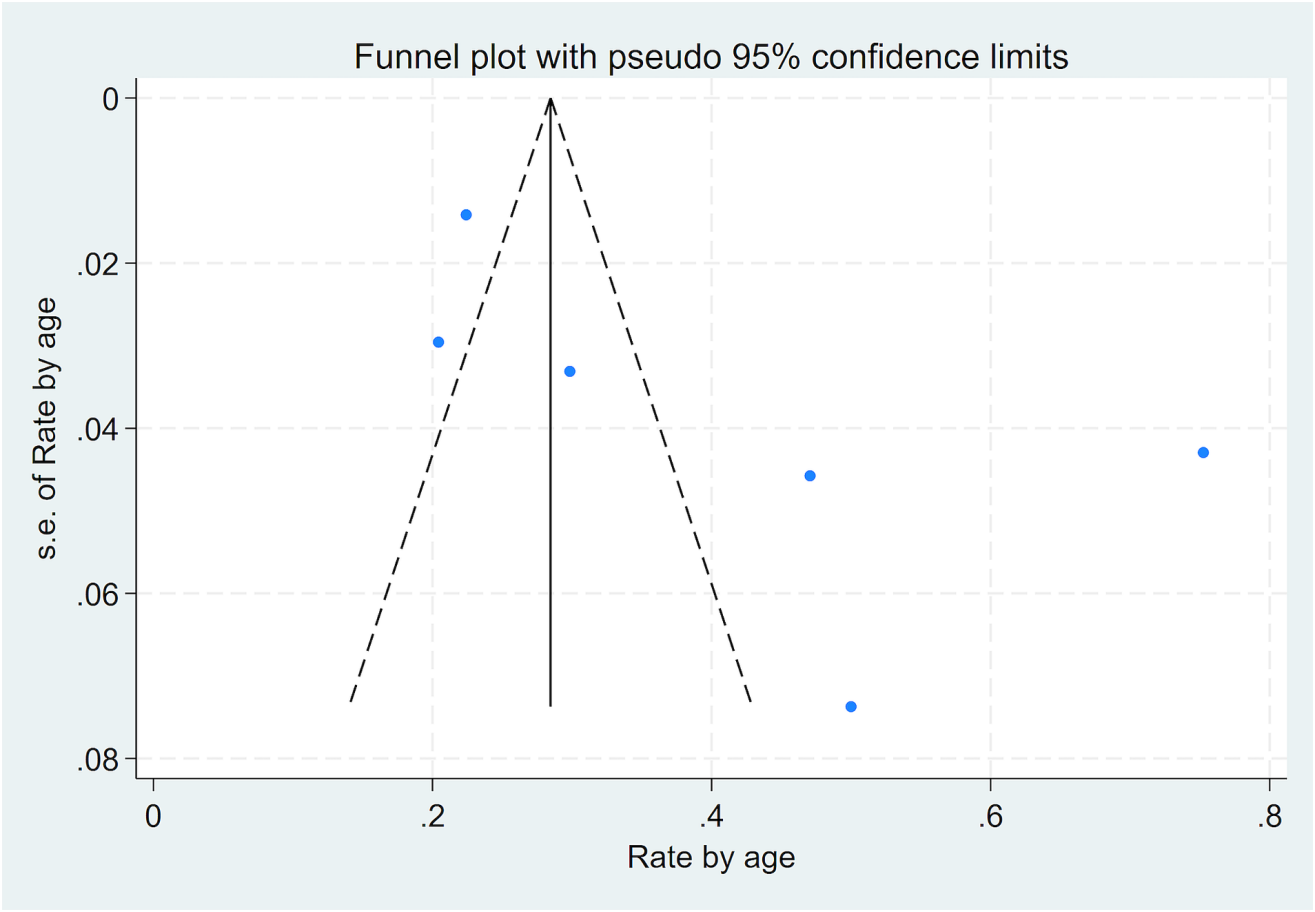
Funnel plot for subgroup analysis by age.

**Figure 9:**
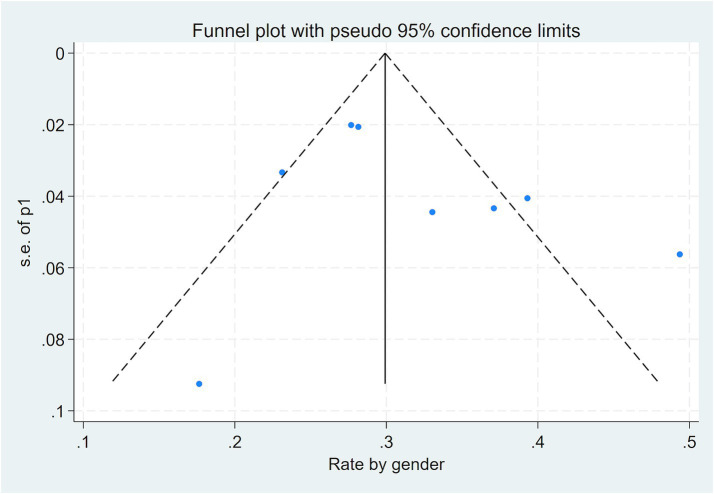
Funnel plot for subgroup analysis by gender.

**Figure 10:**
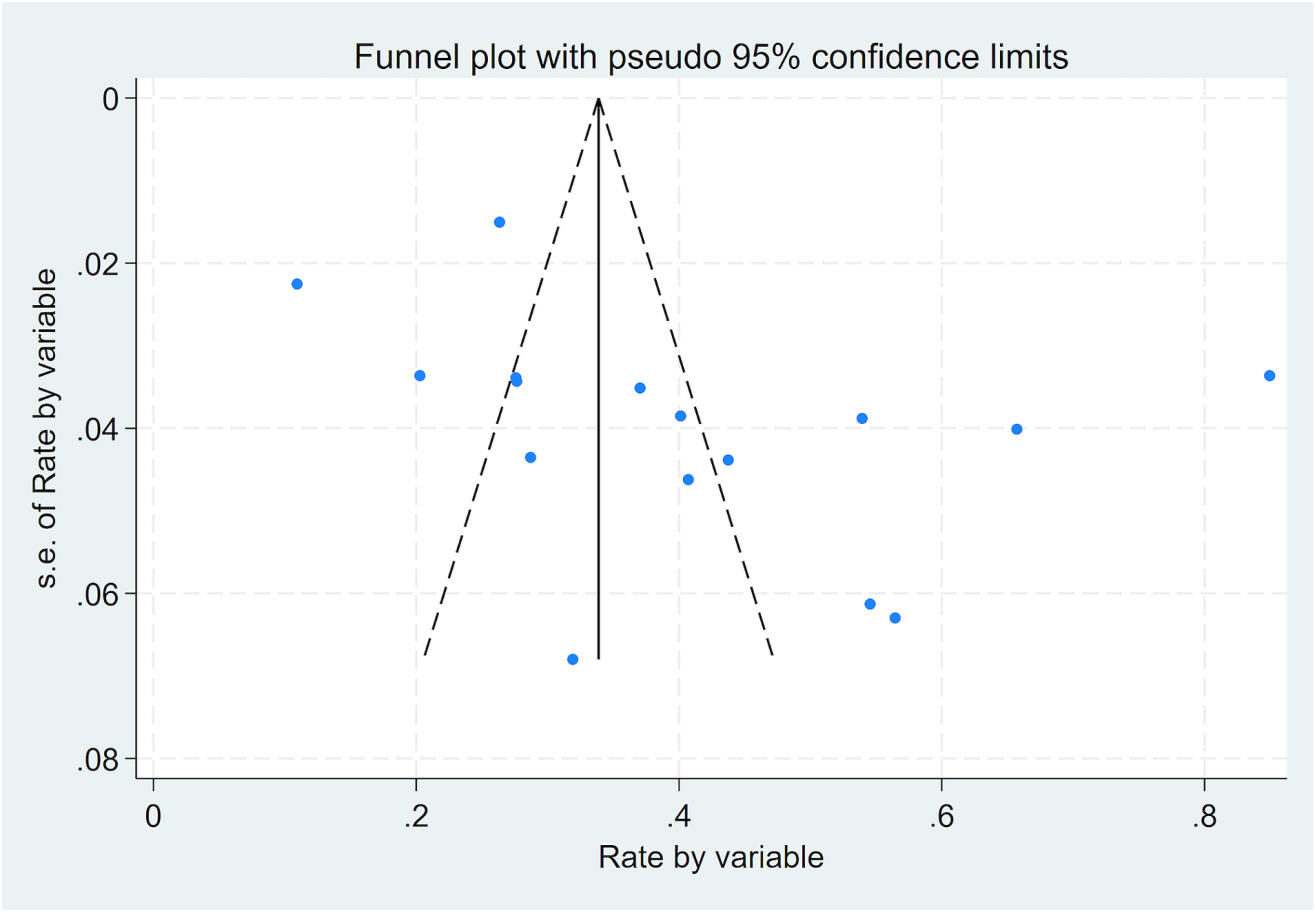
Funnel plot for risk factor subgroups (e.g., alcohol, malnutrition, depression).

Begg’s test results indicated no significant publication bias (*Z* = 1.70, *p* = 0.089, Figure 6). This conclusion was corroborated by visual inspection of the funnel plots. The funnel plot for the overall prevalence analysis demonstrated approximate symmetry, suggesting a low risk of publication bias at the overall level (Figure 7). In the subgroup analyses, the funnel plot for the age subgroup showed asymmetry, indicating potential publication bias specific to this subgroup (Figure 8). In contrast, the funnel plots for the gender subgroup and for subgroups of other variables (alcohol consumption, malnutrition, depression, marital status) exhibited general symmetry, implying a low risk of publication bias in these analyses (Figures 9, 10). It is important to note that, due to the limited number of studies available for some subgroups, these findings should be interpreted with caution.

## 4 Discussion

### 4.1 Meta-analysis of the overall prevalence of CF in older adults with stroke

CF is prevalent among older adults with stroke and is associated with adverse health outcomes (12–17). However, prevalence estimates vary, and data from China remain limited. This meta-analysis of four high-quality studies yielded a pooled CF prevalence of 33% (95% CI: 28–39%), consistent with prior reports (15, 18, 19). Caution is warranted in generalizing this estimate given the small study number and restricted sample representativeness.

The high statistical heterogeneity observed (*I*^2^ = 75.6%) requires careful consideration. A primary source of this heterogeneity is likely the variability in the assessment tools and diagnostic criteria for CF used across the included studies. As detailed in Table 1, the four included studies employed four distinct assessment combinations (e.g., FRAIL/CDR, FRAIL/MMSE, Edmonton/CDR/MMSE, and Fried/MMSE). The lack of a consensus on the optimal tool for post-stroke CF likely resulted in the inclusion of populations with differing severity and characteristics under the broad diagnostic category of “cognitive frailty”. This fundamental methodological variation is a key explanation for the high *I*^2^ value and the disparity in prevalence estimates across studies. Notwithstanding this variability, pooling these operational definitions is methodologically defensible under a random-effects framework, as all studies targeted the same underlying construct using validated instruments. Consequently, our findings underscore an urgent need to develop and validate a unified, stroke-specific CF assessment protocol to ensure the comparability of future research.

### 4.2 Subgroup analysis of CF prevalence in older adults with stroke

Subgroup analyses identified age and alcohol consumption as significant factors associated with the prevalence of CF among older adults with stroke, offering hypothesis-generating insights that may inform future clinical research directions.

#### 4.2.1 Age

Our analysis revealed a significantly higher prevalence of CF in stroke patients aged 80 years or older compared to those aged 60–79 years (*p* < 0.05). This result underscores age as a clinical correlate associated with higher CF prevalence in this population, aligning with the conclusions of prior studies (12–14, 20–22).

Advancing age is associated with physiological changes, including a reduction in neuronal count, weakened synaptic connections, and decreased cerebral blood flow. These changes impair the brain’s capacity for post-stroke recovery (23). Consequently, cognitive reserve diminishes, increasing vulnerability to the co-occurrence of cognitive impairment and physical frailty. Furthermore, older stroke patients often present with multiple chronic comorbidities, such as hypertension, diabetes, and heart disease. The management of these conditions, along with potential side effects of polypharmacy, may exacerbate both cognitive decline and physical frailty, compounding CF risk (24).

In light of these findings, enhanced monitoring and regular assessment of cognitive and physical function may be warranted for older stroke patients. Early detection of CF signs may prompt consideration of targeted interventions, including cognitive training, tailored rehabilitation exercises, and nutritional support (23–25). Implementing such measures may help slow the progression of CF and improve patient quality of life.

#### 4.2.2 Alcohol consumption

Alcohol consumption was associated with significantly higher CF prevalence (*p* < 0.05), consistent with the results reported by Pan et al. (14) but diverges from those of Lin et al. (13). We posit that this discrepancy may be explained by two primary factors. First, the study by Lin et al. (13) had a relatively small sample size (*n* = 236), which may have limited its statistical power to detect a significant association. Second, notable heterogeneity existed in how alcohol consumption was defined and measured across studies. Although most employed a binary classification (yes/no) based on self-report, the specific criteria and thresholds used to define “consumption” varied. This methodological inconsistency could contribute to divergent findings. Therefore, further high-quality studies with standardized definitions of alcohol exposure are necessary to clarify the precise relationship between alcohol consumption and CF in older adults with stroke.

Research indicates that alcohol can cross the blood–brain barrier and exert direct neurotoxic effects on neurons, causing neuronal damage and synaptic dysfunction that negatively impact cognitive function (26). Heavy drinking increases cognitive impairment risk, though a J-shaped relationship with potential protective effects of moderate consumption has been reported in Western populations (27). Long-term heavy alcohol use adversely affects nutritional intake and lifestyle patterns, which may precipitate or exacerbate cerebrovascular events such as cerebral hemorrhage and infarction. These events, in turn, could aggravate both cognitive impairment and physical frailty (28, 29).

Therefore, for older adults with stroke, health education and psychological support targeted at individuals who consume alcohol may warrant consideration. Actively promoting reduction or cessation of alcohol use may help lower the prevalence of CF in this population.

#### 4.2.3 Gender, malnutrition, depression, and marital status

No statistically significant differences in CF prevalence were observed in relation to gender, malnutrition, depression, or marital status among older adults with stroke. The specific findings for each factor are detailed below.

(1) *Gender*: Our analysis found no significant difference in CF prevalence between male and female older adults with stroke, a result consistent with the findings of Lin et al. (13). It is noteworthy that some studies have reported a higher prevalence of CF in female patients compared to males (30, 31). This discrepancy may stem from females’ greater susceptibility to certain psychosocial risk factors, such as hormonal changes and social role transitions. Therefore, further large-scale studies with in-depth mechanistic analyses are needed to clarify the relationship between gender and CF in this population.
(2) *Malnutrition*: The prevalence of CF among older adults with stroke who had malnutrition was 47% (95% CI: 34–60%), which is notably high. This finding underscores the importance of routine nutritional assessment, even in the absence of statistical significance. Malnutrition has been established as an independent risk factor for CF (32). A meta-analysis by Feng et al. (33) indicated that older adults with CF face a 2.77 times higher risk of malnutrition compared to those without CF, with approximately 57% of older CF patients experiencing malnutrition. Older stroke patients are particularly vulnerable to malnutrition due to factors such as dysphagia and reduced appetite. Nutritional deficits may compromise immune function, muscle mass, and bone density, detrimentally affecting both physical and cognitive function. Research suggests that nutritional interventions can slow the progression from CF to dementia in older adults (34). Therefore, providing systematic nutritional support and individualized dietary management for older adults with stroke is crucial to mitigate these risks and potentially improve outcomes.
(3) *Depression*: Although no statistically significant difference in CF prevalence was found between older adults with stroke with and those without depression in this analysis, it is noteworthy that the prevalence among those with depression was 62% (95% CI: 53–71%), a clinically relevant exploratory signal. Depression in older adults with stroke warrants clinical attention, as it affects not only emotional and psychological well-being but may also impair cognitive function. The underlying mechanism may involve neuroendocrine pathways that alter neurotransmitter levels and neural connectivity in the brain (35). Psychological interventions such as cognitive behavioral therapy, mindfulness-based approaches, and cognitive rehabilitation programs have been shown to significantly improve cognitive function and quality of life in stroke patients (36), suggesting a potential avenue for managing modifiable depression-related CF risk. Research specifically examining the depression-CF link in post-stroke populations remains limited.
(4) *Marital status*: This analysis did not identify a statistically significant association between marital status and CF prevalence in older adults with stroke. This finding is inconsistent with the results reported by Lin et al. (13) and Zhao et al. (15). The discrepancy may be attributed to the limited availability of detailed marital status subgroup data in the included literature, which could have affected the analytical precision. Nilaweera et al. (37) followed 12,789 community-dwelling Australians aged ≥70 years and found that adverse life events, particularly spousal bereavement, were associated with increased cognitive impairment risk. Furthermore, research indicates that spouses provide essential emotional support, caregiving assistance, and facilitate social participation, all of which are significant contributors to the recovery process in older stroke patients. Patients with spousal support may be better equipped to manage the challenges and psychological stress associated with stroke, which could positively influence both cognitive and physical function (38).

### 4.3 Limitations of the study

This study provides a systematic assessment of the prevalence of CF in older adults with stroke; however, several limitations must be acknowledged.

(1) The most notable limitation concerns the heterogeneity in the operational definition of CF. The included studies used different assessment tools and diagnostic criteria. This methodological variability is likely a primary source of the observed statistical heterogeneity and complicates the direct comparison of findings across studies. Although a random-effects model was applied to account for this heterogeneity, the small number of studies (*n* = 4) precluded meaningful sensitivity analyses. This further highlights the preliminary nature of the pooled estimate. Future studies should prioritize the development and validation of more consistent, accurate, and standardized assessment tools.
(2) Research in this area remains in an early stage both in China and internationally. The limited number of included studies constrains the robustness of the meta-analysis and reduces the statistical power of the publication bias assessment. Furthermore, all included studies were conducted and published in China, which may limit the generalizability of the findings to other populations and healthcare systems. Additionally, the substantial heterogeneity observed in certain analyses reflects considerable variability across studies. The robustness of some subgroup analyses was further limited by the small number of studies within specific subgroups. Collectively, these factors may affect the precision of the pooled estimates and the wider applicability of the conclusions.
(3) This study relied primarily on data from cross-sectional and cohort studies, which are unable to establish direct causal relationships. Further research is necessary to investigate the underlying mechanisms implicated in CF in this population.

## 5 Conclusion

This study found that the overall prevalence of CF among older adults with stroke in China is 33%, suggesting that CF may be a prevalent condition in older stroke patients and warrants significant clinical attention. However, the generalizability of these results may be limited by the small number of included studies (*n* = 4), their exclusively hospital-based design, and their restricted geographical scope (three Chinese cities), as well as the high statistical heterogeneity observed across studies. Subgroup analysis further identified advanced age and alcohol consumption as significant factors associated with CF prevalence. Furthermore, the notably high point estimates observed in patients with malnutrition (47%) and depression (62%) highlight these conditions as critical clinical characteristics that may require prioritized attention in the management of older adults with stroke. Nonetheless, these subgroup findings should be interpreted cautiously given the limited number of contributing studies and the high overall heterogeneity. These insights may provide direction for the more precise identification of high-risk individuals in clinical practice, pending confirmation in larger, more diverse study populations.

Future studies should prioritize developing and validating a simplified, accurate, and standardized CF assessment toolkit. Such a tool would minimize diagnostic variability and enhance comparability across studies and populations. Additionally, large-scale multicenter studies with substantially larger sample sizes, including international collaborations to incorporate data from underrepresented regions, are needed to validate the cross-cultural adaptability and clinical utility of CF assessment tools. Prospective research should move beyond hospital-based settings to encompass community-dwelling older adults with stroke, thereby capturing a broader and more representative spectrum of disease severity and healthcare access. Community-based cohort studies, in particular, would be valuable for establishing prevalence estimates in non-hospitalized populations and to assess whether the high prevalence observed in current hospital-based research generalizes to milder cases identified in community settings. Leveraging these insights, greater emphasis should be placed on the early identification of CF and on establishing reliable predictive indicators for this condition in older adults with stroke. Advancing these efforts will support the development of more targeted and evidence-based strategies for the prevention and management of CF, ultimately improving health outcomes and quality of life for affected individuals.

## Data Availability

The original contributions presented in the study are included in the article/Supplementary material, further inquiries can be directed to the corresponding authors.
