# Supplementary material for: Prevalence of cognitive frailty in older adults with stroke in China: a systematic review and meta-analysis
Source: Front Neurol. 2026 Jul 8;17:1742673. doi: 10.3389/fneur.2026.1742673 (PMC13389268; doi:10.3389/fneur.2026.1742673)
Supplement: Supplementary file 1 [file Table_1.DOCX]

**Supplementary file 1：Search strategies**

| **PubMed** |
| --- |
| #1: "cognitive frailty"[Title/Abstract] OR "cognitive frail"[Title/Abstract] |
| #2:("mild cognitive impairment"[Title/Abstract] OR "post-stroke cognitive impairment"[Title/Abstract]) AND ("frailty"[Mesh] OR "physical frailty"[Title/Abstract]) AND ("stroke"[Mesh] OR "post-stroke"[Title/Abstract]) |
| #3:("stroke"[Mesh] OR "post-stroke"[Title/Abstract]) AND ("cognitive decline"[Title/Abstract] OR "cognitive dysfunction"[Mesh]) AND frail*[Title/Abstract] |
| **#4:#1 OR #2 OR #3** |
| #5:"Aged"[Mesh] OR "Aged, 80 and over"[Mesh] OR "aged 60"[Title/Abstract] OR "≥60 years"[Title/Abstract] |
| #6:"Cross-Sectional Studies"[Mesh] OR "Cohort Studies"[Mesh] OR prevalence[Title/Abstract] |
| **#7: #4 AND #5 AND #6** |

| **Embase** |
| --- |
| #1:'cognitive frailty':ab,ti |
| #2:('mild cognitive impairment':ab,ti OR 'post stroke cognitive impairment':ab,ti) AND ('frailty'/exp OR 'physical frailty':ab,ti) AND ('stroke'/exp OR 'post stroke'/exp) |
| #3:('stroke'/exp OR 'cerebrovascular accident':ab,ti) AND ('cognitive decline':ab,ti OR 'cognitive dysfunction'/exp) AND frail*:ab,ti |
| **#4:#1 OR #2 OR #3** |
| #5:exp aged/ OR 'aged 60':ab,ti OR '>=60 years':ab,ti |
| #6:exp cross sectional study/ OR exp cohort study/ OR prevalence:ab,ti |
| **#7:#4 AND #5 AND #6** |

| **Web of Science** |
| --- |
| TS=("cognitive frailty" OR "cognitive frail") OR  TS=("mild cognitive impairment" OR "post-stroke cognitive impairment") AND TS=("physical frailty") AND TS=(stroke OR "post-stroke") OR  TS=(stroke OR "cerebrovascular accident") AND TS=("cognitive decline") AND TS=frail* AND  TS=("aged 60" OR "≥60 years" OR elderly) AND  TS=(prevalence OR "cross-sectional" OR cohort) |

| **Cochrane Library** |
| --- |
| #1: 'cognitive frailty':ti,ab,kw |
| #2: ([mh "cognitive dysfunction"] OR "mild cognitive impairment":ti,ab,kw) AND [mh frailty] AND [mh stroke] |
| #3: [mh stroke] AND ("cognitive decline":ti,ab,kw OR [mh "cognitive dysfunction"]) AND frail*:ti,ab,kw |
| **#4:#1 OR #2 OR #3** |
| #5: [mh aged] OR "aged 60":ti,ab,kw |
| #6: [mh "cross-sectional studies"] OR prevalence:ti,ab,kw |
| **#7:#4 AND #5 AND #6** |

| **CBM** |
| --- |
| #1:主题词:认知衰弱/全部树 OR 关键词:认知衰弱 |
| #2:主题词:认知障碍/全部树 AND 主题词:衰弱/全部树 AND 主题词:卒中/全部树 |
| #3:主题词:卒中/全部树 AND (主题词:认知障碍/全部树 OR 主题词:衰弱/全部树) |
| **#4:#1 OR #2 OR #3** |
| #5:主题词:老年人/全部树 OR 关键词:老年人 OR 关键词:高龄 OR 关键词:60岁 |
| #6:主题词:患病率/全部树 OR 主题词:流行病学/全部树 OR 主题词:横断面研究/全部树 |
| #7:**#4 AND #5 AND #6** |

| **CKNI** |
| --- |
| (SU=('认知衰弱') AND SU=('脑卒中'+'卒中'+'中风'+'脑梗死'+'脑出血'))  OR  (SU=('认知障碍'+'轻度认知障碍') AND SU=('衰弱'+'虚弱'+'衰弱综合征') AND SU=('脑卒中'+'卒中'+'中风'))  OR  (SU=('脑卒中'+'卒中'+'中风') AND SU=('认知障碍'+'认知功能下降'+'认知损害') AND SU=('衰弱'+'虚弱'+'衰弱综合征'))  AND SU=('老年人'+'高龄'+'60岁'+'≥60岁')  AND SU=('患病率'+'发病率'+'流行病学'+'横断面研究'+'队列研究') |

| **VIP** |
| --- |
| ((M=认知衰弱 AND M=脑卒中)  OR (M=认知障碍 AND M=衰弱 AND M=脑卒中)  OR (M=脑卒中 AND (M=认知障碍 OR M=认知功能下降) AND (M=衰弱 OR M=虚弱)))  AND (M=老年人 OR M=高龄 OR M=60岁)  AND (M=患病率 OR M=流行病学 OR M=横断面研究) |

| **Wanfang** |
| --- |
| (主题:(认知衰弱) AND 主题:(脑卒中 OR 卒中 OR 中风 OR 脑梗死 OR 脑出血))  OR (主题:(认知障碍 OR 轻度认知障碍) AND 主题:(衰弱 OR 虚弱) AND 主题:(脑卒中 OR 卒中 OR 中风))  OR (主题:(脑卒中 OR 卒中 OR 中风) AND 主题:(认知障碍 OR 认知功能下降 OR 认知损害) AND 主题:(衰弱 OR 虚弱 OR 衰弱综合征))  AND 主题:(老年人 OR 高龄 OR 60岁 OR ≥60岁)  AND (主题:(患病率 OR 发病率 OR 流行病学) OR 文献类型:(横断面研究 OR 队列研究)) |
